# Macedonia in War-Time: Its Country and Inhabitants

**Published:** 1916-09-16

**Authors:** 


### MACEDONIA IN WAR-TIME.

### Its Country and Inhabitants.

By AN OFFICER IN THE R.A.M.C. AND THE AUTHOR OF "NATURE NOTES."

Months have stretched into years since last I sent notes on Nature to The Hospital. Not that I have not seen much to amuse and interest me in the ways of birds and beasts in many fields of war, but distractions are frequent on active service; it is hard to settle to the task.

I am in Macedonia and north of Salonika; that much I may say without giving away the situation, or exposing myself to censorship. The camp is scattered widely about an undulating plain bounded by high hills bare of trees of any size, but in places densely wooded with scrub: chiefly oak and certain thorny bushes. The plain itself is bare and has scattered bushes and much bramble-covered waste amongst its cultivated patches. Farming is primitive. It is hardly the right word here. "Cultivation" is better, for though there are considerable flocks of sheep and goats and herds of cattle, these do not go with the cultivation, seemingly, but belong to a separate industry. The plough is light, of wood, shared with iron and drawn by heavy-bodied, thick-set, short-legged oxen that seem well-kept and resemble in markings and colour Jersey cattle. Water buffaloes, not different from those used in India, are sometimes substituted, but these latter more often haul heavy wooden trains of the most primitive type and piled with enormous loads. The buffaloes also look well-kept. The Macedonian is good to his beast. Though his ideas are liberal as to the burden of an ass, and though he does not count his own weight any addition to the burden, yet the animals seem well-fed and comfortable. I have seen no active ill-treatment of beasts of burden, nor signs of untended galls and sores.

### Slovenly Cultivation.

There are no hedges. The fields are demarked only by the line of furrow, except they be growing vines, then usually a low bank or a low bank with a slight ditch bounds them. Fig-trees grow in the vineyards amongst the grapes. The potential fertility of the country is great, but years of war and uncertainty do not conduce to careful farming. The cultivation is slovenly; the lands dirty; very little manure is used; the crops are very thin. Between the rough patches of crops is much waste land; some that shows no sign of former cultivation, other that bears marks of the plough.

The crops most conspicuous now (I write in August) are maize, melons, and pumpkins. The people are winnowing cereals on the threshing-floors. Rye, wheat, barley, and millet are the chief crops. The corn is trodden by the ox, the ass, and other domestic animals, assisted by a woman sitting on a sort of sledge. The beasts drag the sledge along the threshing-floor across the straw, round and round, to and fro. The combined effect of their sharp hoofs and the crushing of the weight of the sledge forces the grain from the husk and breaks the straw into a rough chaff, which is fed to domestic animals. When a brisk breeze blows the people assemble on the threshing-floors and toss the result of the treading into the air with wooden forks of three or four prongs slightly spaded. The heavy grain falls at once to the floor, the dust is blown away on to the surrounding fields or houses or casual passers-by, the chaff leaps to leeward of the grain. Then the grain is shovelled into a large sieve and sorted into good and bad in separate heaps.

### Characteristics of the Inhabitants.

The people are mixed in kind; Greeks, Turks, and Bulgars. Though they inhabit the same villages they do not intermarry, I am informed, nor do they indulge in racial quarrels. The dress is bright, dirty, and picturesque. Minor differences mark the races, but the common feature is very baggy-seated trousers and a voluminous shawl wound many times round the waist. Such women as are not suckling babies shortly expect to be doing so. As a people they are not lovely. Those that live or work in the river valleys or near the lakes show markedly in their pallid, anæmic complexion the devastating effects of ague.

Since 1912 there has been much shifting of population. The Turks largely left the land when it came under Greek government. The Turks also expelled from Thrace many Greek peasants who had their homes there. Great hardship resulted. The dispossessed Greeks lost their all, and came paupers into the land. They have been allowed to settle on the land left vacant by the Turks, but as they have neither capital, cattle, nor implements they live squalidly in ruinous Turkish houses and eke a scant living from stony hill-sides. An oldish man, fine of face and figure and wearing a dress that, though shabby, showed evidence of better quality than his fellows, attracted my attention in a certain village through which I was riding with an interpreter. Because he was the first fine-looking Greek I had seen, I bade the interpreter question him. He told me that he came from Thrace, where he had been a rich man with wide fields and 8,000 sheep, but the Turks had taken all and turned him out of their land. "And how am I to work to get food now when I am an old man?" he ended his tale.

